# Advisory Committee on Immunization Practices Recommended Immunization Schedule for Children and Adolescents Aged 18 Years or Younger — United States, 2022

**DOI:** 10.15585/mmwr.mm7107a2

**Published:** 2022-02-18

**Authors:** A. Patricia Wodi, Neil Murthy, Henry Bernstein, Veronica McNally, Sybil Cineas, Kevin Ault

**Affiliations:** ^1^Immunization Services Division, National Center for Immunization and Respiratory Diseases, CDC; ^2^Zucker School of Medicine at Hofstra/Northwell and Cohen Children’s Medical Center, New Hyde Park, New York; ^3^Fanny Strong Foundation, West Bloomfield, Michigan; ^4^The Warren Alpert Medical School of Brown University, Providence, Rhode Island; ^5^University of Kansas Medical Center, Kansas City, Kansas.

At its November 2021 meeting, the Advisory Committee on Immunization Practices* (ACIP) approved the Recommended Child and Adolescent Immunization Schedule for Ages 18 Years or Younger—United States, 2022. The 2022 child and adolescent immunization schedule, found on the CDC immunization schedule website (https://www.cdc.gov/vaccines/schedules), summarizes ACIP recommendations, including several changes from the 2021 immunization schedule^†^ on the cover page, tables, and notes. The 2022 child and adolescent schedule also includes a newly created appendix that lists the contraindications and precautions for all vaccine types in the schedule. Health care providers are advised to use the tables, notes, and appendix together. This immunization schedule is recommended by ACIP (https://www.cdc.gov/vaccines/acip) and approved by CDC (https://www.cdc.gov), the American Academy of Pediatrics (https://www.aap.org), the American Academy of Family Physicians (https://www.aafp.org), the American College of Obstetricians and Gynecologists (http://www.acog.org), the American College of Nurse-Midwives (https://www.midwife.org), the American Academy of Physician Associates (https://www.aapa.org), and the National Association of Pediatric Nurse Practitioners (https://www.napnap.org).

ACIP’s recommendations for the use of each vaccine are developed after in-depth reviews of vaccine-related data, including the epidemiology and societal impacts of the vaccine-preventable disease, vaccine efficacy and effectiveness, vaccine safety, quality of evidence, feasibility of program implementation, and economic analyses of immunization policy (*1*). The child and adolescent immunization schedule is published annually to consolidate and summarize updates to ACIP recommendations on vaccination of children and adolescents, and to assist health care providers in implementing current ACIP recommendations. The use of vaccine trade names in this report and in the child and adolescent immunization schedule is for identification purposes only and does not imply specific product endorsement by ACIP or CDC.

For further guidance on the use of each vaccine, health care providers are referred to the respective ACIP vaccine recommendations at https://www.cdc.gov/vaccines/hcp/acip-recs. Providers should be aware that changes in recommendations for specific vaccines can occur between these annual updates to the child and adolescent immunization schedule. If errors or omissions are discovered within the schedule, CDC will post revised versions on the CDC immunization schedule website.^§^ Printable versions of the 2022 child and adolescent immunization schedule and ordering instructions are available on the immunization schedule website. For CDC’s interim clinical considerations for the use of COVID-19 vaccines, health care providers are referred to https://www.cdc.gov/vaccines/covid-19/clinical-considerations/covid-19-vaccines-us.html.

## Changes in the 2022 Child and Adolescent Immunization Schedule

Vaccine-specific changes in the 2022 child and adolescent immunization schedule for children and adolescents aged ≤18 years include new or updated ACIP recommendations for influenza vaccine (*2*), dengue vaccine (*3*), and COVID-19 vaccine (https://www.cdc.gov/vaccines/hcp/acip-recs/vacc-specific/covid-19.html). Changes also include clarification of the recommendations for *Haemophilus influenzae* type b vaccine (Hib); hepatitis A vaccine (HepA); hepatitis B vaccine (HepB); human papillomavirus vaccine (HPV); measles, mumps, and rubella vaccine (MMR); meningococcal serogroups A, C, W, Y vaccine (MenACWY); tetanus, diphtheria, and pertussis vaccine (Tdap); and varicella vaccine (VAR). In addition, a newly created appendix was added that lists the contraindications and precautions for each vaccine type included in the schedule. Following are the changes to the cover page, Tables 1, 2, and 3, the Vaccine Notes, and the Appendix.

### Cover Page

• Dengue vaccine (DENGVAXIA) has been added to the table of vaccine abbreviations and trade names.

• Instructions on how to use the child and adolescent immunization schedule have been updated to include a fifth step asking health care providers to review the appendix, which lists the contraindications and precautions for each vaccine type.

• Instructions on how health care providers can contact CDC with questions and comments about the schedule have been added.

• The section on helpful information was updated to include information on accessing Vaccine Information Statements.

• A QR code directing providers to the immunization schedule website has been added.

### Table 1 (Routine Immunization Schedule)

• The color of the columns for children aged 4–6 years, children aged 11–12 years, and adolescents aged 16 years has been changed from gray to black to align with the color of the other age range columns. The sentence in the table header stating “School entry and adolescent vaccine age groups are shaded in gray” has been deleted.

• **Tdap row:** The overlying text in the column for children aged 11–12 years has been changed from “Tdap” to “1 dose” to be consistent with the format used for other vaccines in the table.

• **HPV row:** The asterisk that was previously present for children aged 9–10 years and its associated descriptive text in the table legend (i.e., “*can be used in this age group”) have been deleted. Instead, the color for children aged 9–10 years has been changed from blue to checked yellow, which is a new color in Table 1. Within the table’s legend, a new checked yellow box has been added, which now indicates that “Recommended vaccination can begin in this age group.”

• **Dengue row:** A new row has been added with the boxes for children and adolescents aged 9–16 years highlighted in yellow to indicate the recommended age for routine dengue vaccination. The overlying text “Seropositive in endemic areas only (see notes)” has been added to the yellow boxes.

### Table 2 (Catch-Up Immunization Schedule)

• **Hib row:** The text for the 4-week minimum interval between doses 2 and 3 has been revised to include recommendations for DTaP-IPV-Hib-HepB (Vaxelis). The text now reads, “if current age is younger than 12 months ***and*** first dose was administered at younger than age 7 months ***and*** at least 1 previous dose was PRP-T (ActHib, Pentacel, Hiberix), Vaxelis, or unknown.” In addition, Hib-HepB (Comvax) was deleted from the text for the 8-week minimum interval between doses 2 and 3 because this vaccine product is no longer available.

• **Dengue row:** A new row has been added for the dengue vaccine outlining the minimum age and minimum interval between doses.

### Table 3 (Immunization by Medical Indication Schedule)

• The definition of severe immunosuppression because of HIV infection has been revised to be consistent with ACIP’s General Best Practice Guidelines for Immunization (*4*) and now reads, “<15% or total CD4 cell count of <200/mm^3^.”

• **Legend:** The text that defines the red box in the table’s legend has been edited for clarity and now reads, “Contraindicated or not recommended—vaccine should not be administered.” In addition, the text that defines the checked yellow box in the table’s legend has been edited and now reads, “Vaccination is recommended, and additional doses might be necessary based on medical condition or vaccine. See Notes.”

• **Dengue:** A new row has been added to outline dengue vaccine recommendations by medical conditions or other indications.

### Notes

• **Additional information:** The text for COVID-19 vaccination has been updated to include a hyperlink to the webpage for CDC’s interim clinical considerations for use of COVID-19 vaccines currently approved or authorized in the United States.

• **Dengue:** This new section was added to provide details for routine dengue vaccination in areas with endemic dengue. In addition, a hyperlink referring health care providers to the latest guidance on areas with endemic dengue and prevaccination laboratory testing is included.

• **Hib:** The note was updated to include the recommendations for routine and catch-up vaccination when DTaP-IPV-Hib-HepB (Vaxelis) is used.

• **HepA:** The note was revised to clarify that the age for routine vaccination is age 12–23 months.

• **HepB:** In the “Special situations” section, the text has been revised to clarify and emphasize the recommendations for postvaccination serology and revaccination.

• **HPV:** In the “Special situations” section, the text for immunocompromising conditions has been revised to clarify that 3 doses should be administered regardless of age at initial vaccination.

• **Influenza:** The note has been updated to reflect the recommendations for the 2021–22 influenza season. The “Special situations” section was condensed by moving information on contraindications and precautions for influenza vaccines to the newly created appendix.

• **MMR:** The note on routine vaccination was updated to include recommendations for use of measles, mumps, rubella, and varicella vaccine (MMRV).

• **MenACWY:** Language was added to the notes regarding the recommendation for simultaneous administration with meningococcal serogroup B vaccine (MenB). The text reads, “MenACWY vaccines may be administered simultaneously with MenB vaccines if indicated, but at a different anatomic site, when feasible.”

• **VAR:** The note has been updated to include recommendations for using MMRV and to clarify that a second dose inadvertently administered after at least a 4-week interval may be counted as a valid dose.

### Appendix (Contraindications and Precautions)

A newly created appendix listing the contraindications and precautions for each vaccine type included in the 2022 child and adolescent immunization schedule has been added. The information in the appendix is adapted from ACIP General Best Practice Guidelines for Immunization (*4*) and ACIP recommendations for use of 2021–22 influenza vaccines (*2*).

## Additional Information

The Recommended Child and Adolescent Immunization Schedule, United States, 2022 is available at https://www.cdc.gov/vaccines/schedules/hcp/imz/child-adolescent.html. The full ACIP recommendations for each vaccine are also available at https://www.cdc.gov/vaccines/hcp/acip-recs. All vaccines identified in Tables 1, 2, and 3 (except diphtheria, tetanus, and acellular pertussis vaccine [DTaP], rotavirus, poliovirus vaccines, and PCV13 [Prevnar 13]) also appear in the Recommended Adult Immunization Schedule for Ages 19 Years or Older, United States, 2022, available at https://www.cdc.gov/vaccines/schedules/hcp/imz/adult.html. The notes and appendix for vaccines that appear in both the child and adolescent immunization schedule and the adult immunization schedule have been harmonized to the greatest extent possible.

## Footnotes

* Recommendations for routine use of vaccines in children and adolescents are developed by ACIP, a federal advisory committee chartered to provide expert external advice and guidance to the CDC director on use of vaccines and related agents for the control of vaccine-preventable diseases in the civilian population of the United States. Recommendations for routine use of vaccines in children and adolescents are harmonized to the greatest extent possible with recommendations made by the American Academy of Pediatrics, the American Academy of Family Physicians, the American College of Obstetricians and Gynecologists, the American College of Nurse-Midwives, the American Academy of Physician Associates, and the National Association of Pediatric Nurse Practitioners. ACIP recommendations approved by the CDC director become agency guidelines on the date published in the *Morbidity and Mortality Weekly Report*. Additional information about ACIP is available at https://www.cdc.gov/vaccines/acip.

† Past immunization schedules are available at https://www.cdc.gov/vaccines/schedules/past.html.

§ CDC encourages organizations to use syndication as a more reliable method for displaying the most current and accurate immunization schedules on an organization's website rather than copying these schedules to their websites. Use of content syndication requires a one-time step that ensures an organization’s website displays current schedules as soon as they are published or revised; instructions for the syndication code are available on CDC’s website (https://www.cdc.gov/vaccines/schedules/syndicate.html). CDC also offers technical assistance for implementing this form of content syndication (requests can be emailed to ncirdwebteam@cdc.gov).
